# The Use of Negative Pressure Wound Therapy for Breast Surgeries: A Systematic Review and Meta-Analysis

**DOI:** 10.1177/22925503251336253

**Published:** 2025-05-20

**Authors:** Tal Levit, Oluwatobi Olaiya, Declan C.T. Lavoie, Ronen Avram, Christopher J. Coroneos

**Affiliations:** 1 Michael G. DeGroote School of Medicine, 3710 McMaster University, Hamilton, Ontario, Canada; 2 Division of Plastic Surgery, Department of Surgery, 3710 McMaster University, Hamilton, Ontario, Canada; 3 Department of Health Research Methods, Evidence, and Impact, 3710 McMaster University, Hamilton, Ontario, Canada

**Keywords:** negative-Pressure wound therapy, vacuum-Assisted closure, negative-Pressure dressings, breast, traitement par pression négative, traitement des plaies, fermeture assistée par le vide, pansements sous pression négative, sein

## Abstract

**Background:** Negative pressure wound therapy (NPWT) following breast surgery has emerged as a promising intervention theorized to reduce complication rates, improve patient-important outcomes, and enhance cost-effectiveness. This systematic review and meta-analysis aims to determine outcomes of NPWT following breast surgery. **Methods:** MEDLINE, Embase, CINAHL, Web of Science, and CENTRAL were searched to include all English-language, peer-reviewed observational and randomized controlled trials (RCTs) investigating NPWT on the breast or donor site among patients undergoing breast surgery. Studies evaluated at least one of the following outcomes: wound dehiscence, surgical site infection (SSI), implant loss, re-operation, re-admission, hematoma, seroma, and skin/wound necrosis. Quality of evidence was assessed with GRADE methodology. **Results: **This review includes 31 studies (eight RCTs, 23 observational) analyzing 3320 patients (4326 breasts). High certainty of evidence indicates decreased risk of wound dehiscence among NPWT patients in RCTs for all NPWT application sites (donor: 0.40; 95%CI 0.21, 0.79; breast: 0.59; 95%CI 0.41, 0.84) and observational trials where NPWT was placed on donor sites (0.64; 95%CI 0.42, 0.98). Some evidence indicates NPWT may reduce SSI, hematoma, seroma, and skin/wound necrosis incidence, however results are uncertain and varied in statistical significance. No effect was identified on rates of breast implant loss, re-operation, and re-admission, although this certainty of evidence is very low. **Conclusions:** Our findings suggest NPWT following breast surgery reduces the risk of wound dehiscence, may have some effect on SSIs, hematoma, seroma, and skin/wound necrosis; and does not demonstrate an effect on rates of implant loss, re-operation or re-admission.

## Résumé

**Historique:** Le traitement des plaies par pression négative (TPN) après chirurgie du sein est devenu une intervention prometteuse qui, en théorie, peut réduire les taux de complications, améliorer des résultats importants pour les patientes et s’avérer plus rentable. Cette revue systématique avec méta-analyse vise à établir les résultats de la TPN après une chirurgie du sein. **Méthodologie:** Une recherche de tous les essais contrôlés randomisés et observationnels, revus par les pairs et en langue anglaise, portant sur la TPN du sein ou d’un site donneur et impliquant des patientes ayant subi une chirurgie du sein a été menée dans les bases de données MEDLINE, Embase, CINAHL, Web of Science et CENTRAL. Les études évaluaient au moins l’un des résultats suivants : déhiscence de la plaie, infection du site chirurgical (ISC), perte de l’implant, réintervention, réhospitalisation, hématome, sérome et nécrose de la peau/plaie. La qualité de la preuve a été évaluée au moyen de la méthodologie GRADE. **Résultats:** Cette revue a inclus 31 études (8 ECR, 23 essais observationnels) portant sur 3320 patients (4326 seins). Une forte certitude de la preuve indique un moindre risque de déhiscence de la plaie parmi les patientes TPN dans les ECR pour tous les sites d’application de la TPN (donneur : 0,40; IC à 95 % : 0,21 à 0,79; sein : 0,59; IC à 95 % : 0,41 à 0,84) et dans les études observationnelles quand la TPN a été placée sur les sites donneurs (0,64; IC à 95 % : 0,42 à 0,98). Certaines données probantes indiquent que la TPN pourrait réduire l’incidence des ISC, hématomes, séromes et de la nécrose cutanée/de la plaie. Toutefois, ces résultats ne sont pas certains et ont une significativité statistique variable. Aucun effet n’a été identifié sur les taux de perte des implants, de réintervention et réhospitalisation, bien que le degré de certitude de cette preuve soit très faible. **Conclusions:** Nos constatations suggèrent que la TPN après chirurgie du sein réduit le risque de déhiscence de la plaie et peut avoir un certain effet sur les ISC, les hématomes, les séromes et la nécrose cutanée/de la plaie; en revanche, elles ne font pas apparaître d’effet sur les taux de perte d’implant, de réintervention ou de réhospitalisation.

## Introduction

Breast surgeries are among the most frequently performed plastic surgery procedures.^1^ Despite recent advances in surgical technique and postoperative management, complication rates are up to 30% in some subgroups.^2,3^ The morbidity of these complications varies; common complications include wound dehiscence, surgical site infections (SSIs), hematomas, seromas, and necrosis.^4^ Experiencing postoperative complications compounds overall outcomes, with significantly impaired patient recovery, increased healthcare costs, and substantial psychological impact.^5,6^

Interventions capable of decreasing these complications are important, both in improving patient outcomes and healthcare system costs. Negative pressure wound therapy (NPWT), has been shown to reduce the rate of incision and wound related complications across several surgical specialties.^7^ NPWT facilitates wound healing by applying sub-atmospheric pressure to the wound site, stabilizing the wound environment, promoting drainage to minimize edema and inflammation, and stimulating cellular proliferation.^8^ NPWT has also been found to be cost effective for applications such as chronic wound care, although the evidence on cost-effectiveness for surgical incisions remains uncertain.^9–11^

Previous reviews assessed the use of NPWT for a variety of different surgical fields, including orthopedic, vascular, and plastics.^12–15^ However, there is no comprehensive, up-to-date systematic review and meta-analysis synthesizing all available published literature regarding NPWT for breast surgeries. The most recent relevant systematic review on this topic suggests decreased complication rates associated with NPWT use but is limited by a low number of included studies, inappropriate combination of study designs in analysis, and lack of relevant subgroups.^16^ The last point is especially significant as the scope of breast surgeries varies widely, with significant differences between applying NPWT on a surgical site incision for a mastectomy compared to on the abdominal donor site of a breast reconstruction. This gap underscores the need for a rigorous synthesis of the available evidence to evaluate the efficacy and applicability of NPWT for breast surgeries, thereby informing clinical practices and policies.

## Methods

This systematic review and meta-analysis was conducted and reported in accordance to the methods outlined in the PRISMA (Preferred Reporting Items for Systematic Reviews and Meta-Analyses) guideline.^17^ A protocol for this review was registered in the PROSPERO.^18^

### Eligibility Criteria

Articles were included if they met the following criteria: (1) randomized controlled trials (RCTs) or observational trials (2) investigated the use of NPWT for any breast procedure in adults (>18 years), (3) were published in a peer reviewed journal and the full text was available in English. The exclusion criteria included: reviews, conference abstracts, case studies, case series, letters to the editor, non-human studies, and non-English language studies.

### Search Strategy

A search was conducted on July 10, 2023 across the following databases: MEDLINE, Embase, CINAHL, Web of Science, and Cochrane Central Register of Controlled Trials (CENTRAL). Trial registries including ClinicalTrials.gov (http://clinicaltrials.-gov/), and International Clinical Trials Registry Platform Search Portal (http://apps.who.int/trialsearch/) were searched and cross referenced with the collected publications. References of included studies and relevant previous reviews were scrutinized for any studies that might have been missed by the search. A trained medical librarian collaborated in the creation of a search strategy (see Supplemental Material 1, which outlines the literature search strategy used).

### Study Selection and Data Collection Process

Identified studies underwent independent evaluation in duplicate by two authors utilizing the Covidence review software.^19^ Titles and abstracts meeting the inclusion and exclusion criteria advanced to the full-text screening. Disagreements were resolved with consensus, or senior author input. Data extraction from the included studies was performed independently by two authors into a standardized pilot-tested spreadsheet. Extracted outcomes included: wound dehiscence, surgical site infection, implant loss, re-operation, re-admission, hematoma, seroma, skin/wound necrosis, device failure/couldn’t complete therapy, and use of intravenous (IV) antibiotics. The primary authors’ definition of these outcomes was accepted. There were no constraints on the duration of the study's follow-up.

### Risk of Bias and Quality of Evidence Appraisal

Two authors independently assessed the risk of bias using the Cochrane Risk of Bias 2 (RoB 2) tool^20^ for RCTs, and the Cochrane Risk Of Bias In Non-randomised Studies - of Interventions (ROBINS-I) tool^21^ for observational trials. Items from the RoB 2 and ROBINS-I were extracted into a pre-arranged Excel file, and risk of bias plots were generated using the robvis visualization web tool.^22^ Disagreements were resolved through discussion or by consensus with a blinded senior author. The quality of evidence of each outcome was determined according to the criteria specified in the Grading of Recommendations, Assessment, Development and Evaluations (GRADE) Handbook.^23^ This methodology evaluates certainty based on factors such as risk of bias, inconsistency, indirectness, imprecision, and publication bias. Each outcome was assigned a level of certainty (high, moderate, low, or very low) based on the synthesis of evidence from included studies. Language used to describe the findings reflects the GRADE approach. The corresponding GRADE summary of findings table was created using the GRADEpro GDT software.^24^

### Outcomes and Reporting

The primary outcomes of this study were the effects of NPWT on wound dehiscence, with secondary outcomes including surgical site infection, implant loss, re-operation, re-admission, hematoma, seroma, and skin/wound necrosis. All outcome data were collected and analyzed as categorical outcomes. Events were reported either on a per-patient or a per-breast basis, with incidence being calculated compared to the total number of the original authors preferred value. All studies providing data on an outcome were included in the analysis, and if three or more studies contributed to an outcome domain, a meta-analysis was performed. RCTs and observational trials were analyzed separately due to the different levels of evidence produced.

Post hoc analysis was conducted on two subgroups, as most trials involved NPWT application either at the donor site after flap surgeries (eg, breast reconstruction with a DIEP or latissimus dorsi flap) or on the breast itself following non-flap breast surgeries (eg, mastectomy, reduction mammaplasty, or breast reconstruction with tissue expanders). These subgroups were categorized as the ‘donor site’ and ‘breast site,’ respectively.

### Statistical Analysis and Reporting

This meta-analysis used an inverse-variance random effects model for pooled effects estimates. A risk ratio with a 95% confidence interval (CI) was calculated for discrete outcomes and a p-value of <.05 was considered statistically significant. Data were analyzed and reported in accordance with protocols outlined in the Cochrane Handbook.^25^ Study heterogeneity was assessed across studies using the chi-squared test and I^2^ statistic. Interpretation of the I^2^ value as defined by the Cochrane Handbook is as follows: 0–40% ‘might not be important’, 30%–60% ‘may represent moderate heterogeneity’, 50%–90% ‘may represent substantial heterogeneity’, and 75%–100% ‘considerable heterogeneity’.^25^ Study characteristics and extracted data were organized in tables and presented along with narrative summaries as described in the Cochrane Handbook for systematic reviews and meta-analyses.^25^ Certainty of evidence is presented in a GRADE summary of findings table as outlined in the GRADE Handbook.^23^

## Results

**Figure 1:**
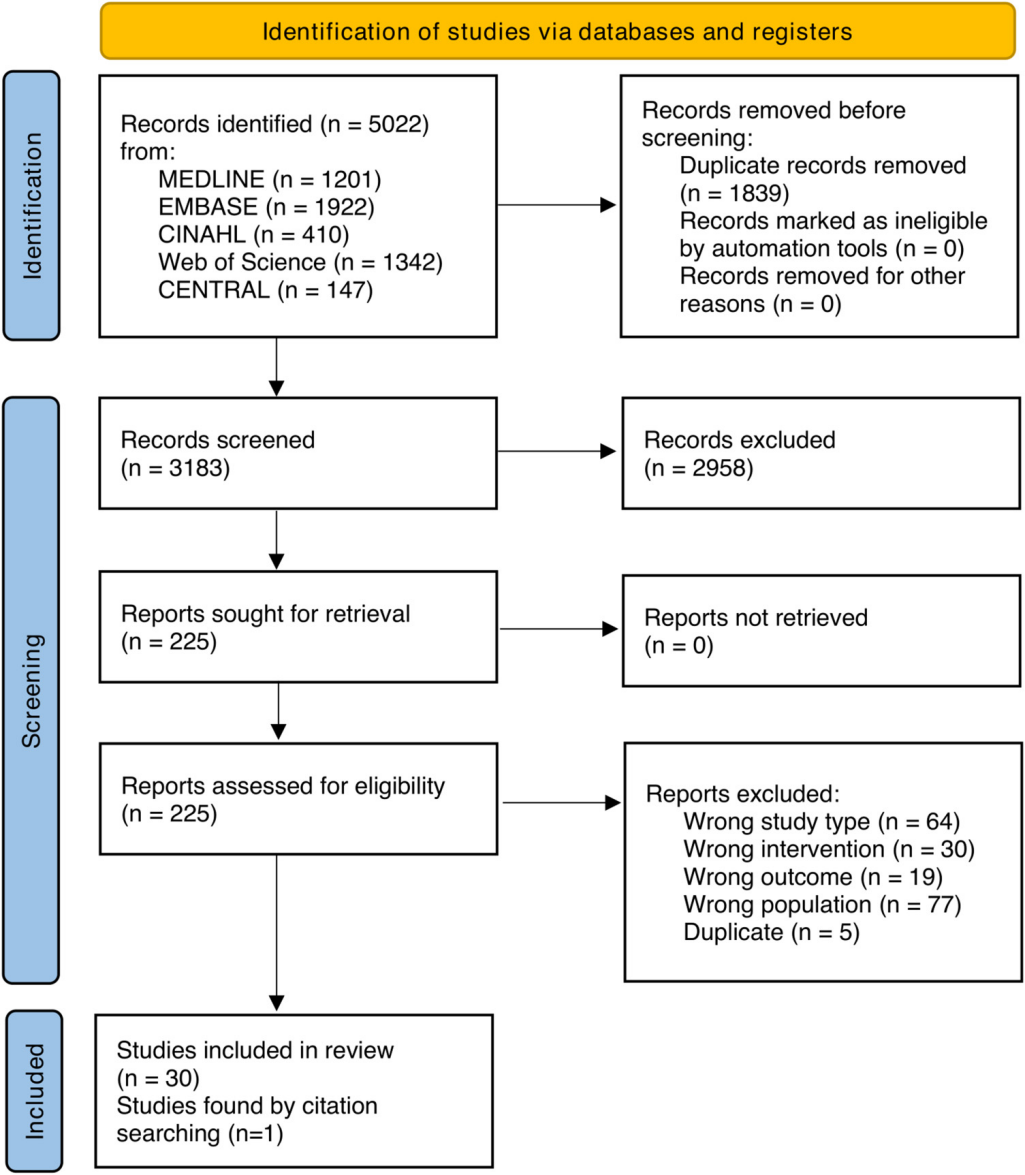
A figure of the PRISMA (preferred reporting items for systematic reviews and meta-analyses) 2020 flow diagram.

The full study selection process is presented in the PRISMA flow chart (Figure 1). After removal of duplicates, 3183 records were identified and screened. We performed a full-text review of 225 reports of which 30 were included^26–55^ with one additional article^56^ discovered through citation searching. Of these, eight are RCTs^33,35,42,43,47,52,53,56^ and 23 are observational trials.^26–32,34,36–41,44–46,48–51,54,55^ Several articles such as Holt 2015^57^ and Gabriel 2016^58^ fit most inclusion criteria but were excluded due to unsuitable study design. Rates of device failure, patients being unable to complete therapy, and use of IV antibiotics were not reported in any of the included studies. 20 studies^27,28,30–34,36,38–44,47,48,50,51,54^ included patients undergoing breast reconstruction and 11^26,29,35,37,45,46,49,52,53,55,56^ included general non-reconstructive breast surgeries. Of the breast reconstruction articles, 9^27,32–34,36,39,40,47,48^ were implant-based with NPWT applied on breast sites, and 11^28,30,31,38,41–44,50,51,54^ were flap-based involving NPWT on donor sites. No articles assessing flap-based reconstructions applied NPWT to breast sites. Thus, 11 articles^28,30,31,38,41–44,50,51,54^ were assigned to the donor subgroup, and 20^26,27,29,32–37,39,40,45–49,52,53,55,56^ were assigned to the breast subgroup. The characteristics of included studies can be found in Supplemental Material 2.

### Risk of Bias

The risk of bias among the included studies is shown in Supplemental Material 3. Five RCTs^33,35,43,52,53^ (two articles assessing reduction mammaplasty, one mastectomy, one DIEP- or PAP-based reconstruction, and one including a variety of breast surgeries) exhibited some concerns for bias arising from the randomization process and the outcome measurement domain due to challenges in blinding. Additionally, no articles provided a predetermined plan in their protocols or trial registries, introducing some concerns for bias. Three RCTs^42,47,56^ (one each assessing mastectomy, mastectomy with immediate implant-based reconstruction, and DIEP-based reconstruction) had high risk of bias due to missing outcome data or bias in the selection of the reported result due to omission of certain reporting data. Among observational trials, all except De Rooij 2021^29^ (mastectomy) had an overall serious concern for risk of bias. Almost none of the articles had robust statistical methods to control for confounding in the study population, and similar to RCTs, blinding was impossible or extremely difficult to perform.

### Wound Dehiscence

**Figure 2:**
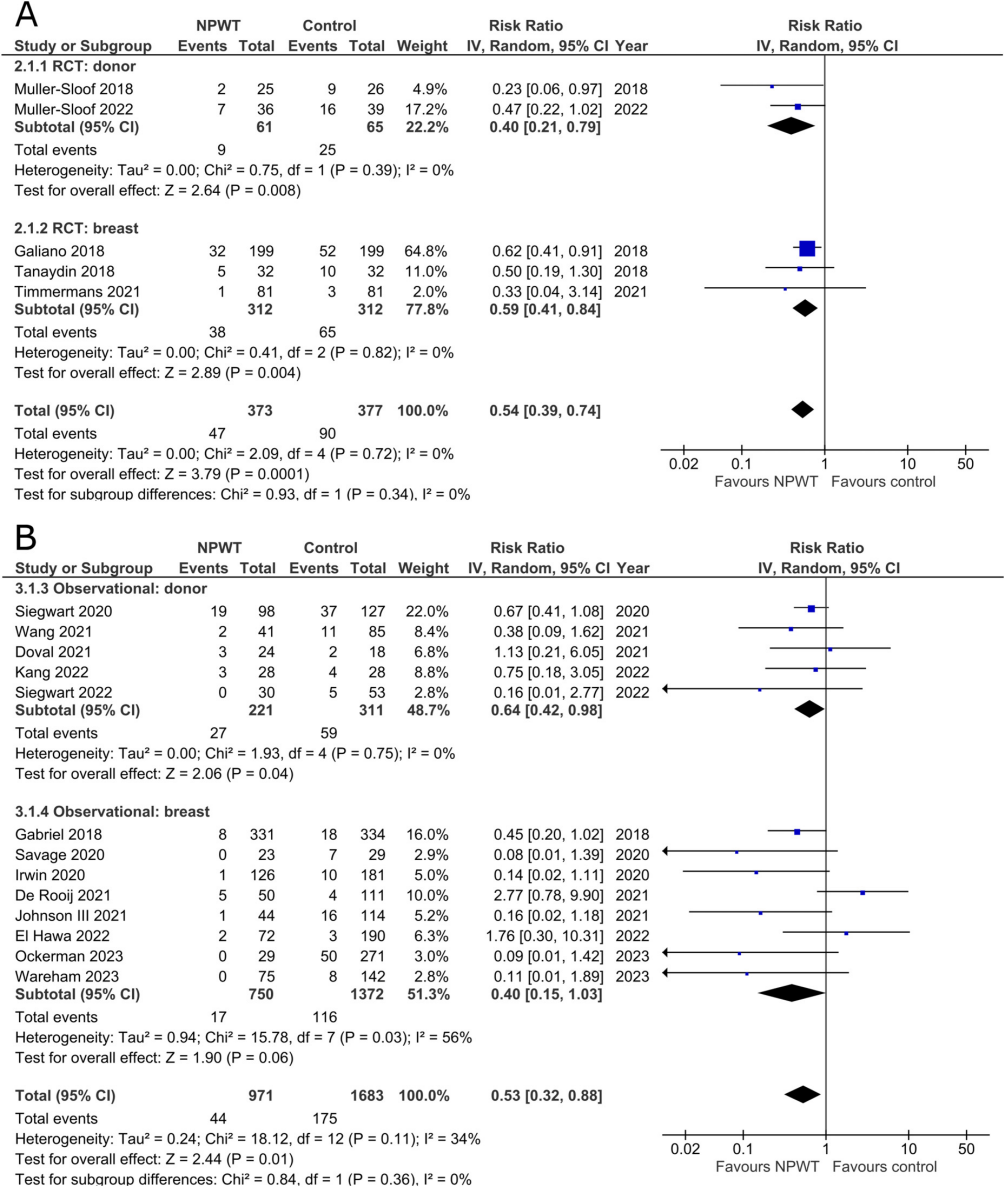
**A:** A forest plot displaying meta-analysis results for wound dehiscence among included randomized controlled trials. (NPWT: Negative pressure wound therapy. RCT: Randomized controlled trial. IV: Inverse variance. CI: Confidence interval). **B:** A forest plot displaying meta-analysis results for wound dehiscence among included observational trials. (NPWT: Negative pressure wound therapy. IV: Inverse variance. CI: Confidence interval).

Incidence of wound dehiscence was reported by five RCTs^35,42,43,52,53^ (two DIEP- or PAP-based reconstruction, two reduction mammaplasty, and one mastectomy) and 13 observational studies^26,29,30,34,36–38,45,49–51,54,55^ (two mastectomy, one mastectomy with immediate implant-based reconstruction, one expander-based reconstruction, three DIEP-, one TMG-, one abdominal-based free flap reconstruction, two reduction mammaplasty, one lumpectomy, and one breast conservation surgery with volume displacement or replacement). Pooled results can be found in Figure 2A and B. Among RCTs there is a statistically significant risk reduction for patients who were treated with NPWT compared to control in both donor (RR 0.40; 95%CI 0.21, 0.79; p < .01) and breast (RR 0.59; 95%CI 0.41, 0.84; p < .01) subgroups with no evidence of heterogeneity (I^2 ^= 0%). In observational trials there is a statistically significant decreased risk of wound dehiscence with NPWT in the donor subgroup (RR 0.64; 95%CI 0.42, 0.98; p = .04; I^2 ^= 0%), and a non-statistically significant decrease (RR 0.40; 95%CI 0.15, 1.03; p = .06; I^2 ^= 56%) in the breast subgroup.

### Surgical Site Infection

**Figure 3:**
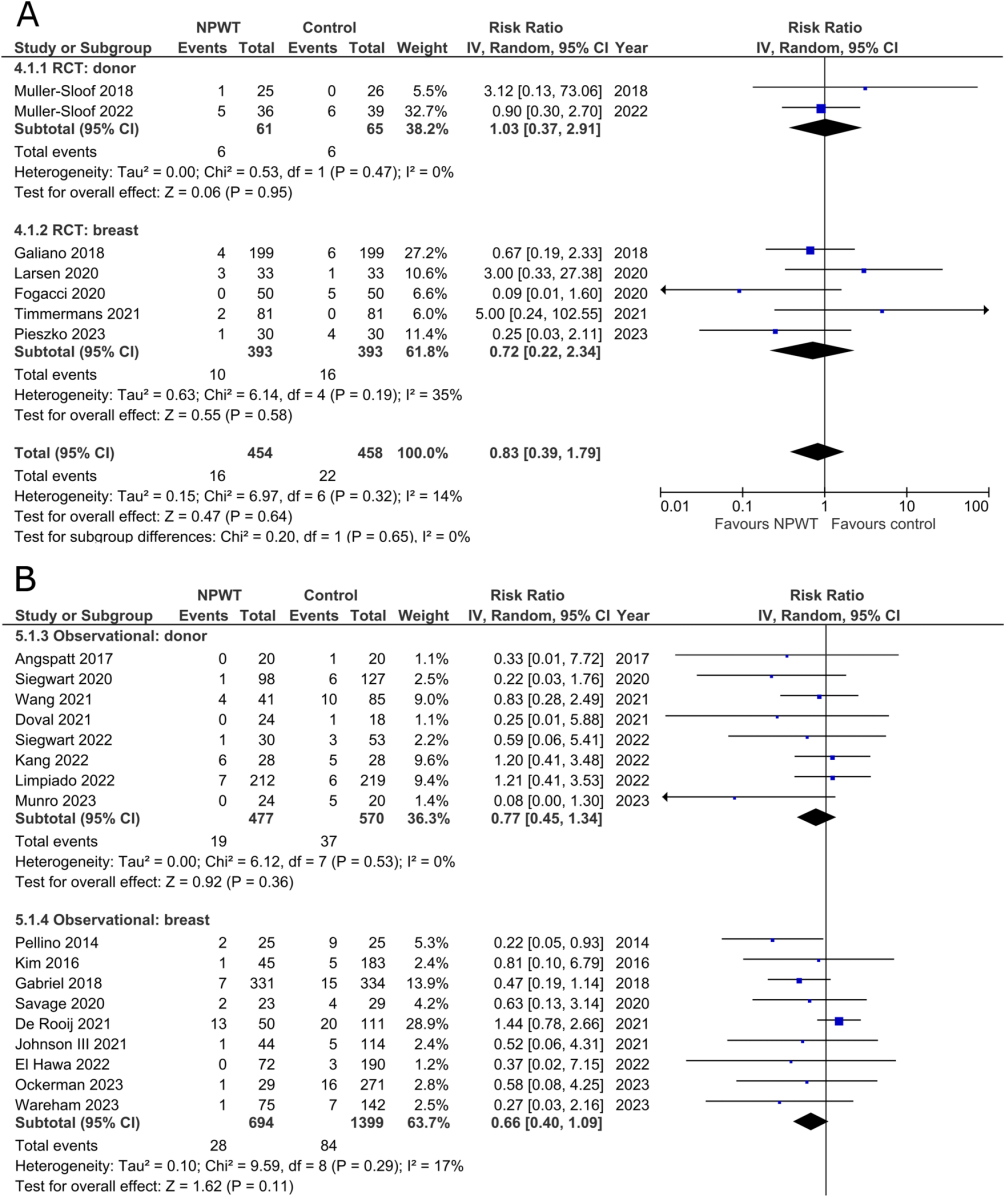
**A:** A forest plot displaying meta-analysis results for surgical site infection among included randomized controlled trials. (NPWT: Negative pressure wound therapy. RCT: Randomized controlled trial. IV: Inverse variance. CI: Confidence interval). **B:** A forest plot displaying meta-analysis results for surgical site infection among included observational trials. (NPWT: Negative pressure wound therapy. IV: Inverse variance. CI: Confidence interval).

Seven RCTs^33,35,42,43,47,53,56^ (two DIEP- or PAP-based reconstruction, two mastectomy, one mastectomy with immediate implant-based reconstruction, one reduction mammaplasty, and one including a variety of breast surgeries) and 17 observational studies^26,28–30,34,37–39,41,44–46,49–51,54,55^ (two mastectomy, two expander-based reconstruction, four DIEP-, one TMG-, one latissimus dorsi-, two abdominal-based free flap reconstruction, two reduction mammaplasty, one lumpectomy, one breast conservation surgery with volume displacement or replacement, and one including a variety of breast surgeries) reported the number of surgical site infections among patients (Figure 3A and B). Risk of SSIs does not differ significantly between NPWT and control in both donor (RR 1.03; 95%CI 0.37, 2.91; p = .95; I^2 ^= 0%) and breast (RR 0.72; 95%CI 0.22, 2.34; p = .58; I^2 ^= 35%) subgroups within RCTs. Observational trials again reveal no statistically significant difference in risk of SSIs when comparing NPWT to control (donor [RR 0.77; 95%CI 0.45, 1.34; p = .36; I^2 ^= 0%] and breast [RR 0.66; 95%CI 0.40, 1.09; p = .11; I^2 ^= 17%]).

### Breast Implant Loss

**Figure 4:**
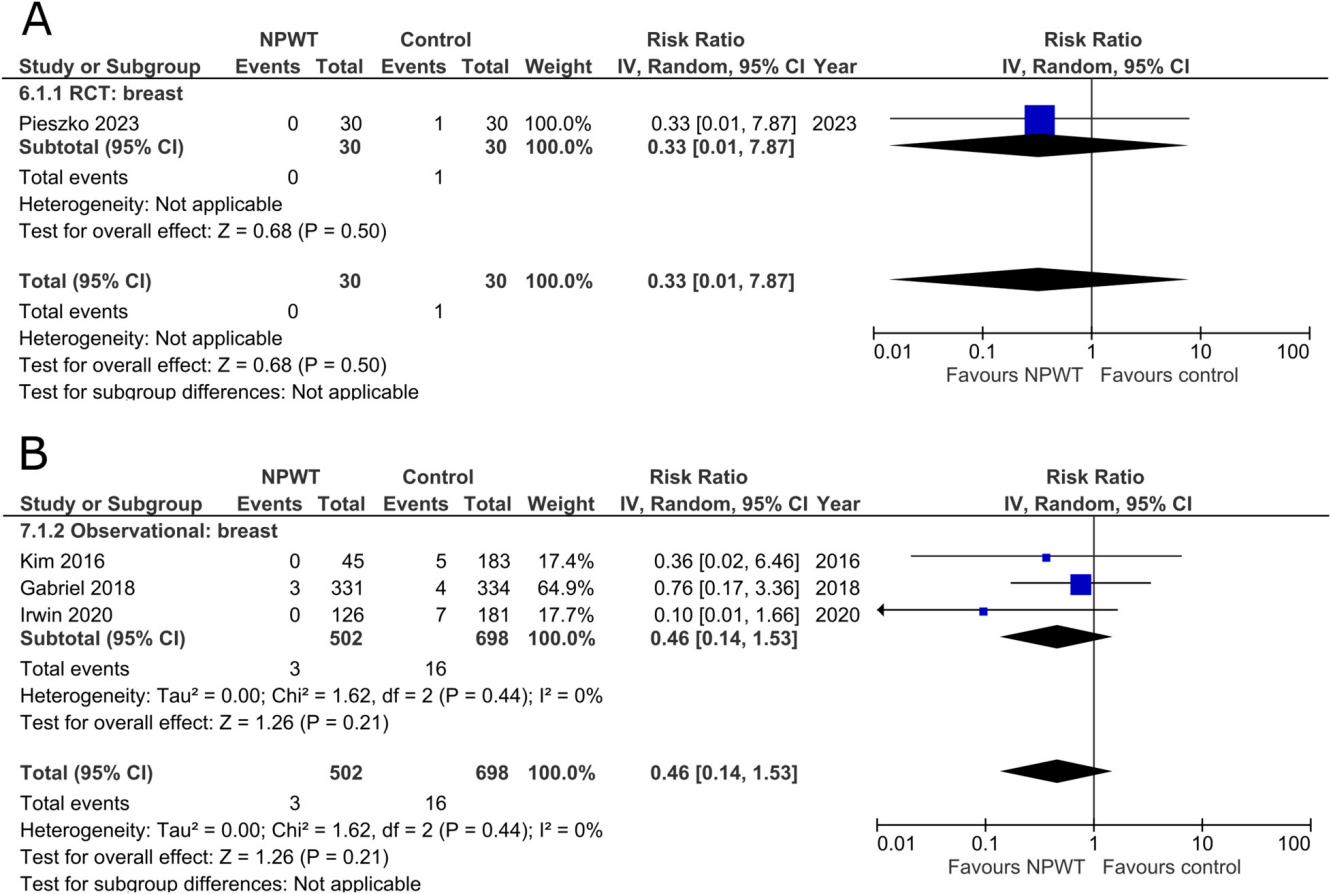
**A:** A forest plot displaying meta-analysis results for breast implant loss among included randomized controlled trials. (NPWT: Negative pressure wound therapy. RCT: Randomized controlled trial. IV: Inverse variance. CI: Confidence interval). **B:** A forest plot displaying meta-analysis results for breast implant loss among included observational trials. (NPWT: Negative pressure wound therapy. IV: Inverse variance. CI: Confidence interval).

Rates of implant loss were reported in only one RCT^47^ (mastectomy with immediate implant-based reconstruction) and three observational studies^34,36,39^ (two expander-based reconstruction and one mastectomy with immediate implant-based reconstruction), all of which included application of NPWT on the breast site. There is no statistically significant difference in risk of implant loss between the NPWT and control group. The RCT describes a risk ratio of 0.33 (95%CI 0.01, 7.87; p = .50), and observational trials have a pooled risk ratio of 0.46 (95%CI 0.14, 1.53; p = .21; I^2 ^= 0%) (Figure 4A and B).

### Re-Operation

**Figure 5:**
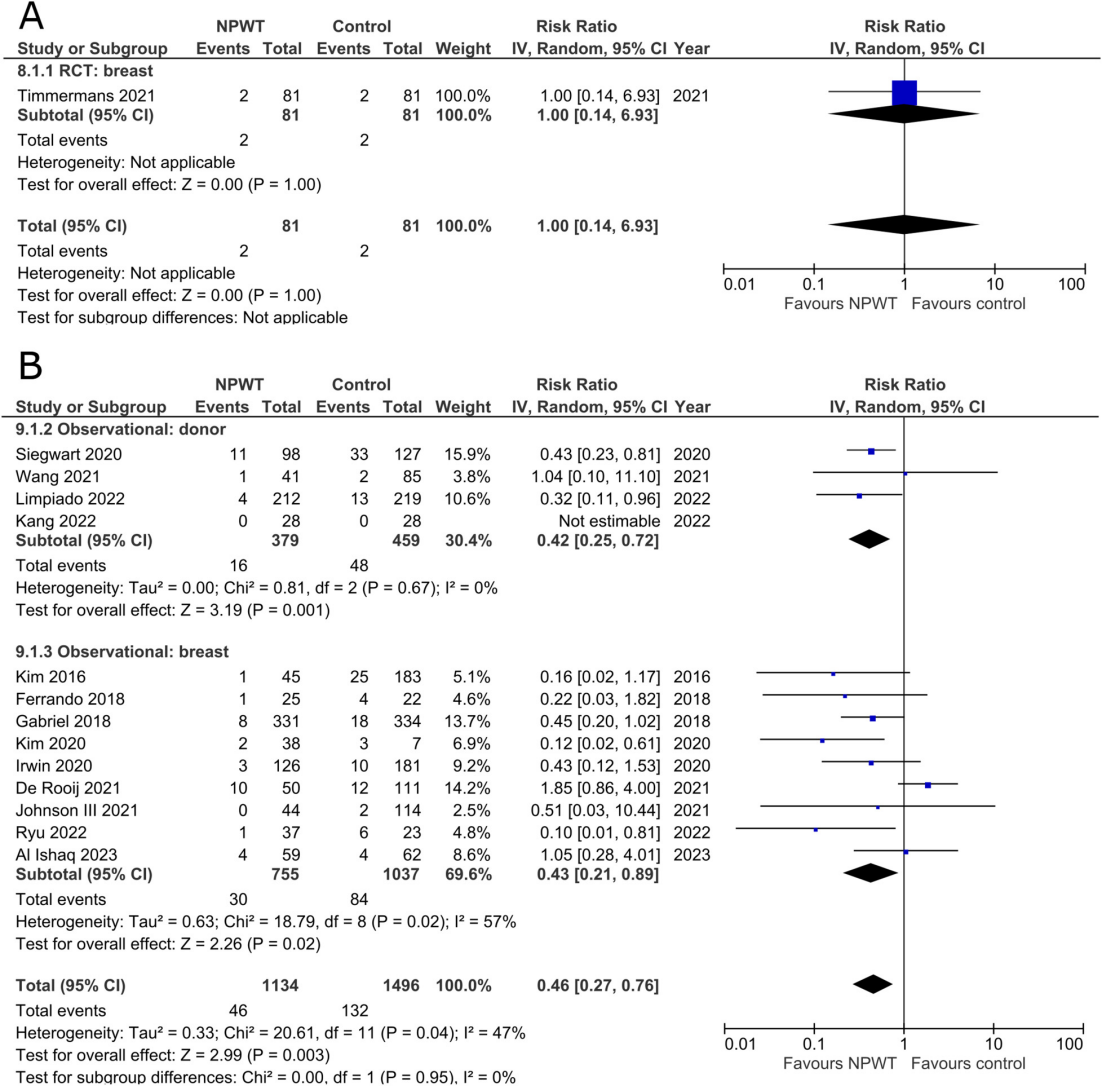
**A:** A forest plot displaying meta-analysis results for re-operation among included randomized controlled trials. (NPWT: Negative pressure wound therapy. RCT: Randomized controlled trial. IV: Inverse variance. CI: Confidence interval). **B:** A forest plot displaying meta-analysis results for re-operation among included observational trials. (NPWT: Negative pressure wound therapy. IV: Inverse variance. CI: Confidence interval).

One RCT^53^ (mastectomy) and 13 observational studies^27,29,32,34,36–41,48,50,54^ (one mastectomy, three mastectomy with immediate implant-based reconstruction, two expander-based reconstruction, two DIEP-, two abdominal-based free flap reconstruction, one reduction mammaplasty, and two including a variety of breast surgeries) reported the risk for re-operation (Figure 5A and B). The RCT, in the breast subgroup, reports equal rates of re-operations between NPWT and control. Among observational trials, a decreased risk of undergoing a re-operation is observed across both donor (RR 0.42; 95%CI 0.25, 0.72; p < .01; I^2 ^= 0%) and breast (RR 0.43; 95%CI 0.21, 0.89; p = .02; I^2 ^= 57%) subgroups.

### Re-Admission

**Figure 6:**
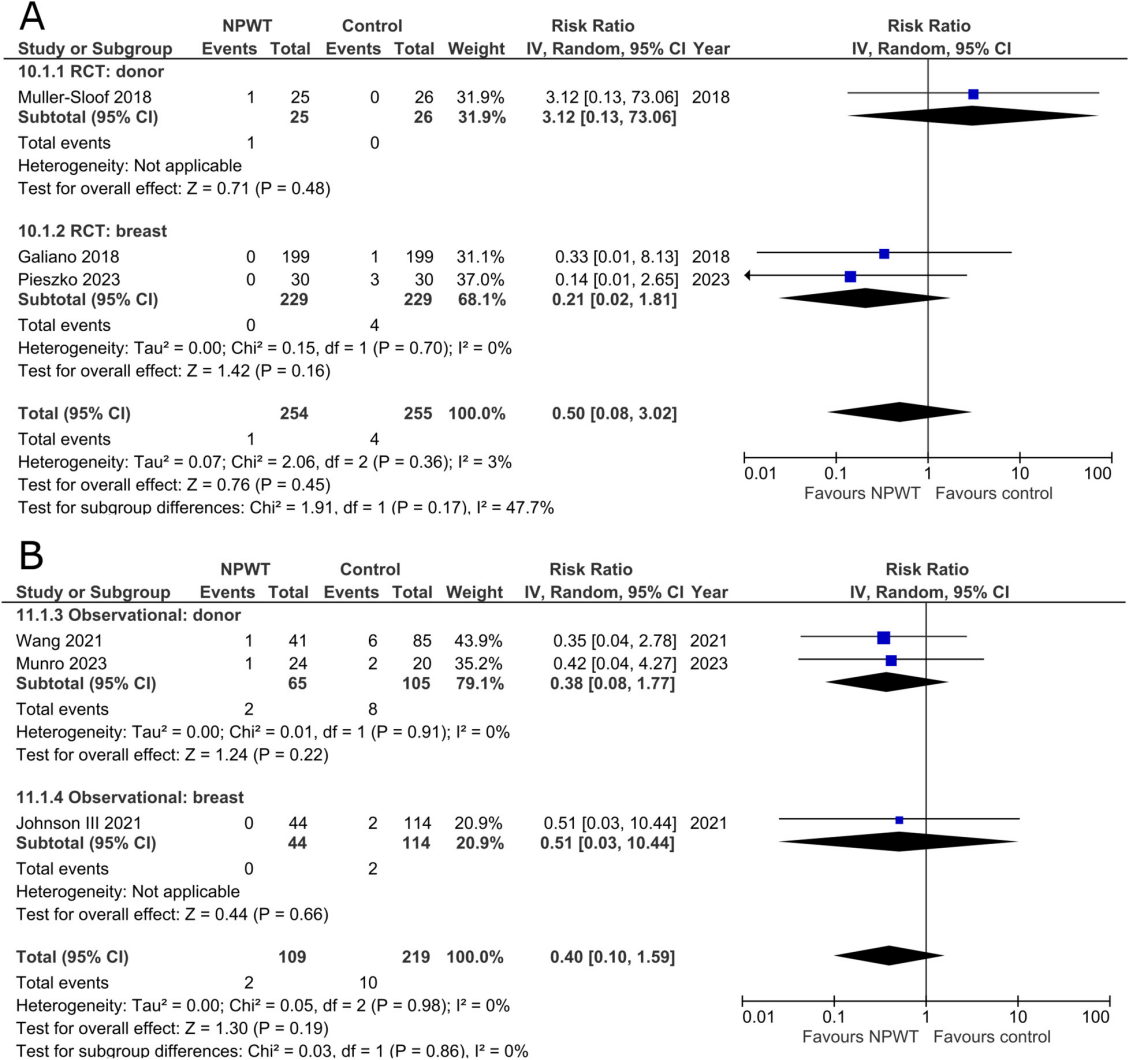
**A:** A forest plot displaying meta-analysis results for re-admission among included randomized controlled trials. (NPWT: Negative pressure wound therapy. RCT: Randomized controlled trial. IV: Inverse variance. CI: Confidence interval). **B:** A forest plot displaying meta-analysis results for re-admission among included observational trials. (NPWT: Negative pressure wound therapy. IV: Inverse variance. CI: Confidence interval).

Three RCTs^35,43,47^ (one DIEP- or PAP-based reconstruction, one mastectomy with immediate implant-based reconstruction, and one reduction mammaplasty) and three observational studies^37,44,54^ (Two DIEP-based reconstruction and one reduction mammaplasty) reported the number of patients that were re-admitted following postoperative discharge. All the included studies fail to demonstrate a difference in risk of re-admission between patients who received NPWT compared to control (Figure 6A and B).

### Hematoma

**Figure 7:**
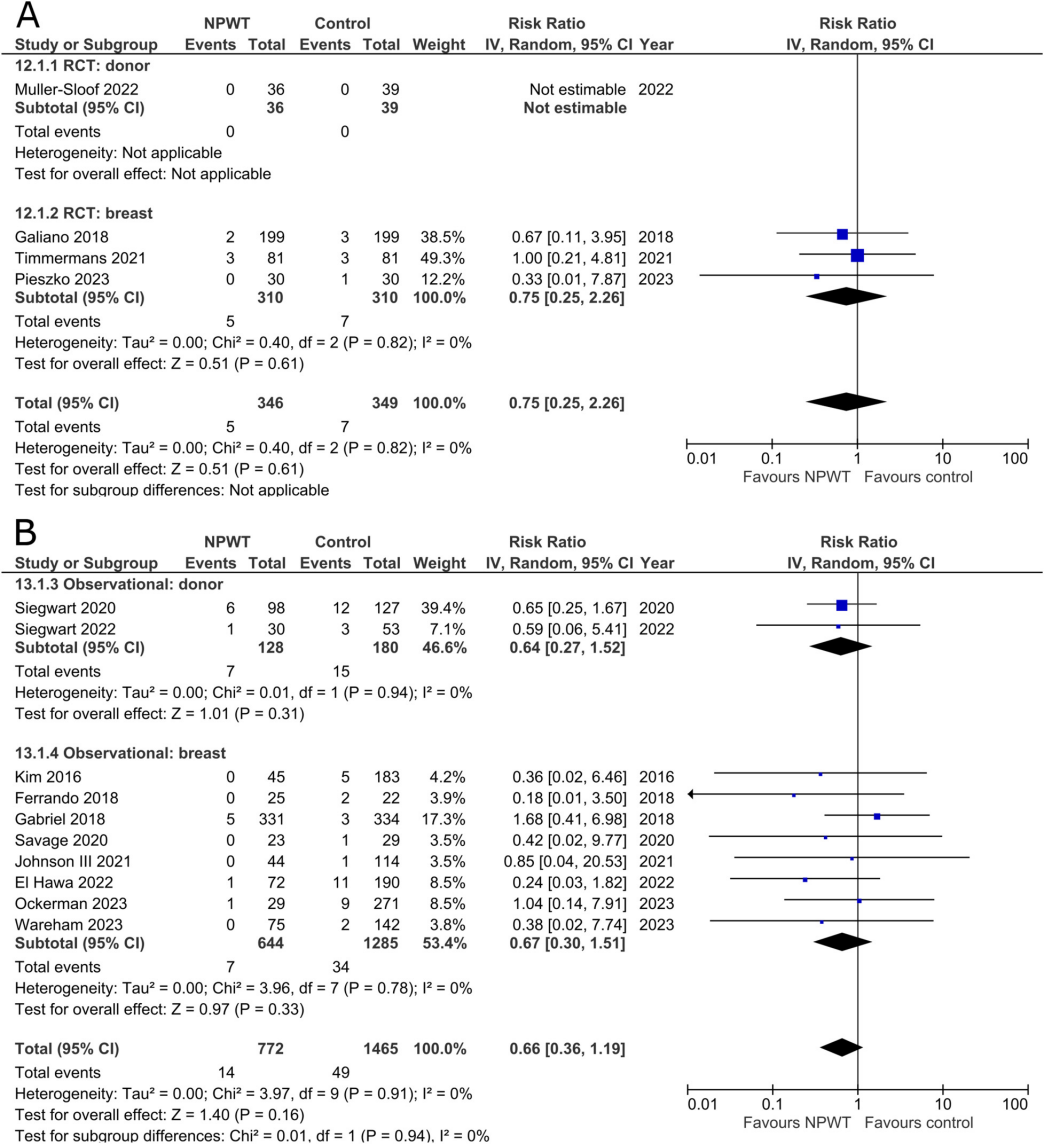
**A:** A forest plot displaying meta-analysis results for hematoma among included randomized controlled trials. (NPWT: Negative pressure wound therapy. RCT: Randomized controlled trial. IV: Inverse variance. CI: Confidence interval). **B:** A forest plot displaying meta-analysis results for hematoma among included observational trials. (NPWT: Negative pressure wound therapy. IV: Inverse variance. CI: Confidence interval).

Hematomas were reported by four RCTs^35,42,47,53^ (one each of DIEP-based reconstruction, mastectomy, mastectomy with immediate implant-based reconstruction, and reduction mammaplasty) and 10 observational studies^26,32,34,37,39,45,49–51,55^ (one mastectomy, two expander-based reconstruction, one TMG-, one abdominal-based free flap reconstruction, two reduction mammaplasty, one lumpectomy, one breast conservation surgery with volume displacement or replacement, and one including a variety of breast surgeries). The RCT in the donor subgroup had no instances of hematomas in either NPWT or control, while the RCTs in the breast subgroup demonstrated no statistically significant difference between NPWT and control for hematoma development (RR 0.75; 95%CI 0.25, 2.26; p = .61; I^2 ^= 0%). Observational trials similarly do not find a statistically significant reduction in hematoma incidence between NPWT and control (donor [RR 0.64; 95%CI 0.27, 1.52; p = .31; I^2 ^= 0%] and breast [RR 0.67; 95%CI 0.30, 1.51; p = .33; I^2 ^= 0%]) (Figure 7A and B).

### Seroma

**Figure 8:**
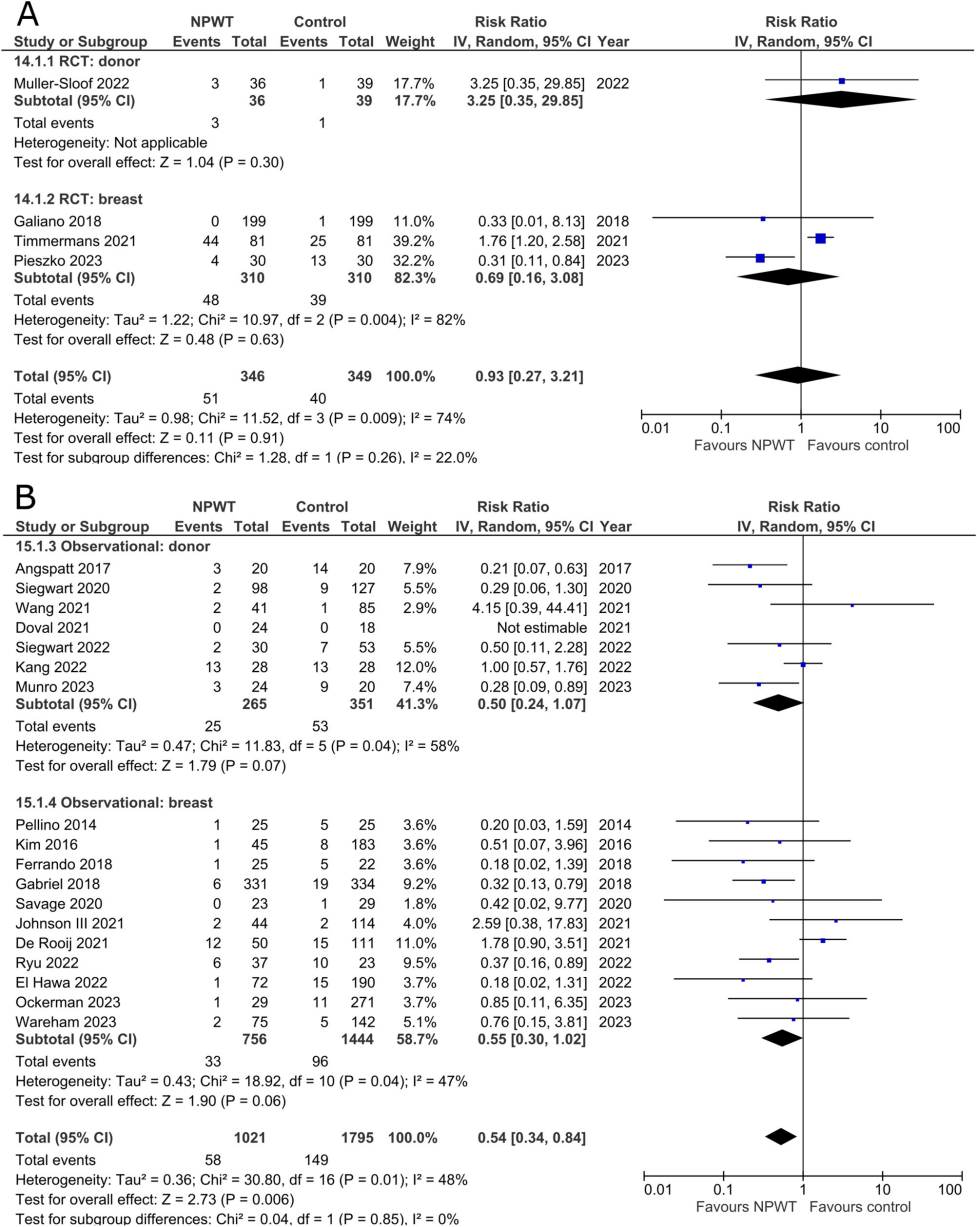
**A:** A forest plot displaying meta-analysis results for seroma among included randomized controlled trials. (NPWT: Negative pressure wound therapy. RCT: Randomized controlled trial. IV: Inverse variance. CI: Confidence interval). **B:** A forest plot displaying meta-analysis results for seroma among included observational trials. (NPWT: Negative pressure wound therapy. IV: Inverse variance. CI: Confidence interval).

Seroma was reported by four RCTs^35,42,47,53^ (one each of DIEP-based reconstruction, mastectomy, mastectomy with immediate implant-based reconstruction, and reduction mammaplasty) and 18 observational studies^26,28–30,32,34,37–39,44–46,48–51,54,55^ (two mastectomy, two mastectomy with immediate implant-based reconstruction, one expander-based reconstruction, four DIEP-, one TMG-, one latissimus dorsi-, one abdominal-based free flap reconstruction, two reduction mammaplasty, one lumpectomy, one breast conservation surgery with volume displacement or replacement, and two including a variety of breast surgeries) (Figure 8A and B). Pooled results from RCTs show no statistically significant difference in seroma risk between NPWT and control groups for both the donor (RR 3.25; 95%CI 0.35, 29.85; p = .30) and breast (RR 0.69; 95%CI 0.16, 3.08; p = .63; I^2 ^= 82%) subgroups. Among observational studies, there is no statistically significant difference in risk between the treatment arms for both donor (RR 0.50; 95%CI 0.24, 1.07; p = .07; I^2 ^= 58%) and breast (RR 0.55; 95%CI 0.30, 1.02; p = .06; I^2 ^= 47%) subgroups.

### Skin/Wound Necrosis

**Figure 9:**
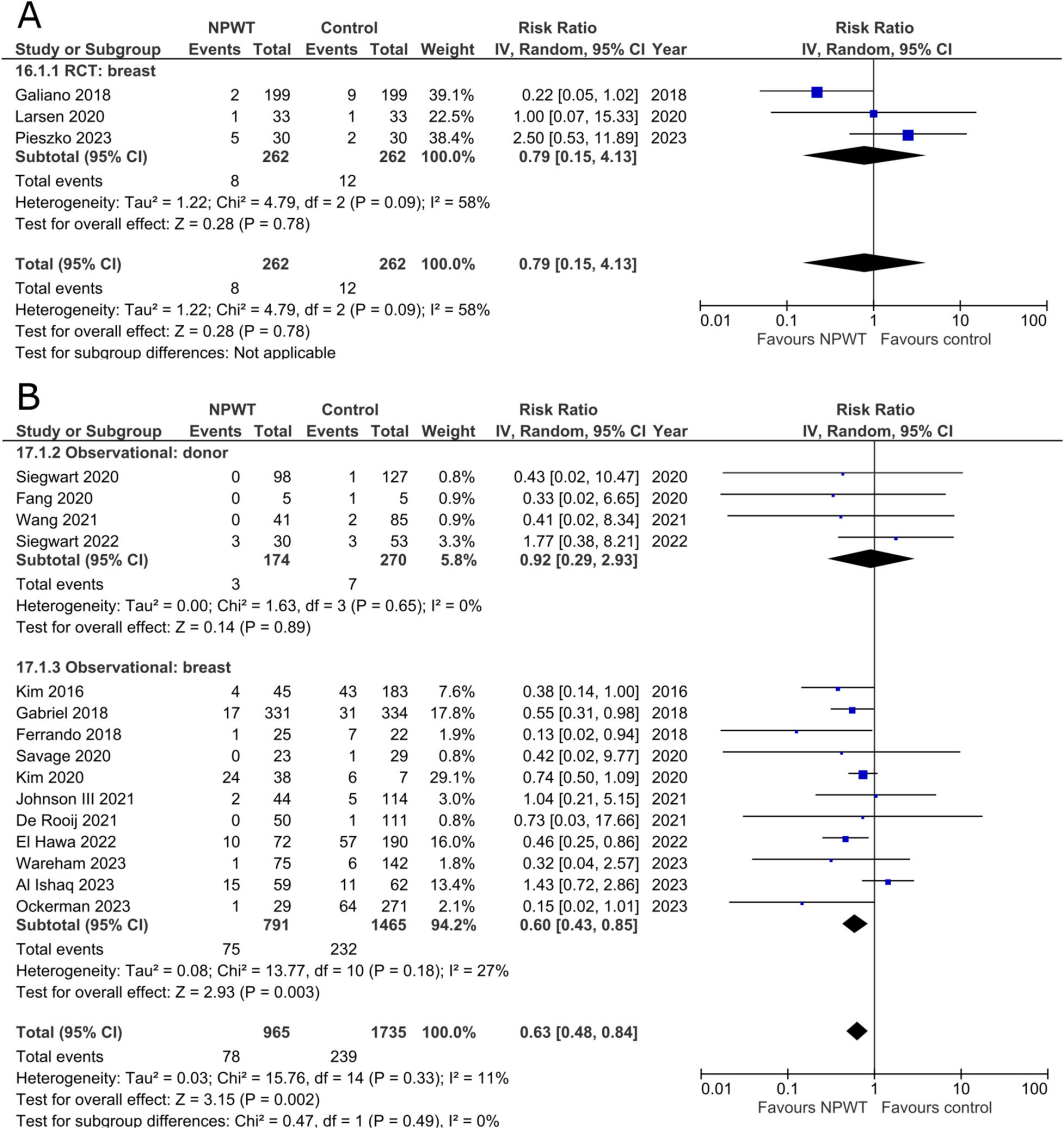
**A:** A forest plot displaying meta-analysis results for skin/wound necrosis among included randomized controlled trials. (NPWT: Negative pressure wound therapy. RCT: Randomized controlled trial. IV: Inverse variance. CI: Confidence interval). **B:** A forest plot displaying meta-analysis results for skin/wound necrosis among included observational trials. (NPWT: Negative pressure wound therapy. IV: Inverse variance. CI: Confidence interval).

Three RCTs^35,47,56^ (one each of mastectomy, mastectomy with immediate implant-based reconstruction, and reduction mammaplasty) and 15 observational studies^26,27,29,31,32,34,37,39,40,45,49–51,54,55^ (two mastectomy, one mastectomy with immediate implant-based reconstruction, two expander-based reconstruction, two DIEP-, one TMG-, one abdominal-based free flap reconstruction, two reduction mammaplasty, one lumpectomy, one breast conservation surgery with volume displacement or replacement, and two including a variety of breast surgeries) described incidence of skin/wound necrosis (Figure 9A and B). Our pooled analysis of three RCTs in the breast subgroup fails to demonstrate a statistically significant difference between treatment arms (RR 0.79; 95%CI 0.15, 4.13; p = .78; I^2 ^= 58%). Observational studies in the donor subgroup find no difference in risk of skin/wound necrosis between NPWT and control (RR 0.92; 95%CI 0.29, 2.93; p = .89; I^2 ^= 0%), while observational studies in the breast subgroup report a relative risk of 0.60 for patients assigned to NPWT (95%CI 0.43, 0.85; p < .01; I^2 ^= 27%).

### GRADE Quality of Evidence

All outcomes in this review were included in the GRADE certainty of evidence summary of findings table. Only RCTs were included in the synthesis due to their higher level on the hierarchy of evidence.^59^ The certainty of evidence was determined to be high for wound dehiscence due to the consistent, direct, and precise result of the data synthesis. Surgical site infections, hematoma, and skin/wound necrosis outcomes remain limited by imprecision of results and thus had a moderate certainty of evidence. Similarly, rates of heterogeneity among seroma subgroups ranged from moderate to high, and thus certainty of evidence is downgraded to moderate. Rates of breast implant loss, re-operation, and re-admission have a very low number of events, with the latter additionally having high heterogeneity, and are thus rated as very low certainty of evidence. The full summary of findings table can be found in Supplemental Material 4.

## Discussion

This study demonstrates that NPWT reduces the risk of wound dehiscence compared to conventional dressings across all types of breast surgery. The decrease is statistically significant in both RCTs and observational trials for flap-based reconstructions involving NPWT placement on the donor site, and among RCTs investigating implant-based reconstructions and general non-reconstructive breast surgeries where NPWT is applied on the breast. This finding is substantial as wound dehiscence represents a particularly challenging post-operative complication. Dehiscence is often associated with SSIs and can lead to delayed healing, increased healthcare costs, and considerable patient distress.^60^ Therefore, preventing its occurrence is advantageous from both a patient important outcome and economic standpoint.^61^ The potential for NPWT being used to prevent postsurgical breast and donor site wound dehiscence is promising and warrants strong consideration in clinical settings.

For surgical site infections, hematoma, seroma, and skin/wound necrosis, pooled risk ratios were often lower with the use of NPWT. However, results varied between subgroups, were often not statistically significant, and suffered from imprecision due to the relatively low number of captured events. Although there may be an effect of NPWT on these outcomes, the quality of evidence is not high according to the GRADE framework. Thus, these outcomes were described as “potential effects” rather than definitive conclusions, based on the language used in GRADE guidelines. These results must be interpreted with their limitations, and clinical recommendations have uncertainty.

Analysis of outcomes such as breast implant loss, re-operation, and re-admission had very low certainty of evidence as they were heavily limited by low number of included studies and low frequency of events that occurred within the study population. Further research needs to be conducted to discern whether application of NPWT affects their incidence.

Besides low frequency of events for certain outcomes, there could be other explanations for why results varied between subgroups. One potential reason for these discrepancies could be due to differences in study populations and surgical interventions. For example, the demographics and associated comorbidities of patients undergoing flap-based breast reconstruction are likely dissimilar to those undergoing reduction mammaplasties. Additionally, the postoperative recovery course for free flap surgeries is different compared to procedures such as implant-based reconstructions and mastectomies. Furthermore, application of NPWT to breast incision sites has a different set of considerations and risk profile compared to that of a donor site. Thus, the authors believed that creating separate subgroups based on these distinctions would provide the most clinically relevant and applicable results.

In practical use, the potential benefits discussed in this paper must be balanced against the possible drawbacks of NPWT, including increased costs and risks of skin irritation from improper application. In Canada, accessibility to NPWT devices remains a challenge, though this is improving with the development of smaller, more mobile, and less costly devices that may help overcome these barriers.

This paper represents the largest and most comprehensive review on NPWT use for breast surgeries to date. With many included studies, the real effects of NPWT use on breast surgery can be better estimated. Several outcomes had low heterogeneity, indicating high consistency of findings among studies for those outcomes, which enhances the generalizability and clinical relevance of these results. Other outcomes displayed moderate to high heterogeneity, decreasing confidence that those results are universally applicable across different patient populations and surgical settings. A limitation to consider is the relatively large number of observational trials in this review. Although eight RCTs were analyzed, more trials would increase the confidence in our results.

Overall, this systematic review suggests strong evidence that NPWT leads to decreased risk of wound dehiscence following breast surgery. There is potentially some evidence that NPWT could reduce frequency of surgical site infections, hematoma, seroma, and skin/wound necrosis, however this is uncertain. For outcomes such as rates of breast implant loss, re-operation, and re-admission the effect is unclear. These findings align with systematic reviews from other fields, which describe that application of NPWT may lead to decreased complication rates for certain outcomes.^12,13^ Therefore, this review summarizes the potential benefits associated with NPWT use and highlights the need for further research, especially in the form of well-designed RCTs, to comprehensively assess the effect of NPWT for breast surgery.

## Conclusion

Available evidence in the literature appears to suggest that negative pressure wound therapy is associated with statistically significantly lower incidence of wound dehiscence in all types of breast surgeries. The certainty of this evidence is high and can be used to inform clinical decision making. There could be some effect on rates of surgical site infections, hematoma, seroma, and skin/wound necrosis, however this is uncertain. There was no effect identified from NPWT use on occurrence of breast implant loss, re-operations or re-admissions. Further high-quality RCTs need to be conducted to finalize the true effects of NPWT on preventing complications for breast surgeries.

## Supplemental Material

sj-docx-1-psg-10.1177_22925503251336253 - Supplemental material for The Use of Negative Pressure Wound Therapy for Breast Surgeries: A Systematic Review and Meta-Analysis Supplemental material, sj-docx-1-psg-10.1177_22925503251336253 for The Use of Negative Pressure Wound Therapy for Breast Surgeries: A Systematic Review and Meta-Analysis by Tal Levit, Oluwatobi Olaiya, Declan C.T. Lavoie, Ronen Avram and Christopher J. Coroneos in Plastic Surgery

sj-docx-2-psg-10.1177_22925503251336253 - Supplemental material for The Use of Negative Pressure Wound Therapy for Breast Surgeries: A Systematic Review and Meta-Analysis Supplemental material, sj-docx-2-psg-10.1177_22925503251336253 for The Use of Negative Pressure Wound Therapy for Breast Surgeries: A Systematic Review and Meta-Analysis by Tal Levit, Oluwatobi Olaiya, Declan C.T. Lavoie, Ronen Avram and Christopher J. Coroneos in Plastic Surgery

sj-tif-3-psg-10.1177_22925503251336253 - Supplemental material for The Use of Negative Pressure Wound Therapy for Breast Surgeries: A Systematic Review and Meta-Analysis Supplemental material, sj-tif-3-psg-10.1177_22925503251336253 for The Use of Negative Pressure Wound Therapy for Breast Surgeries: A Systematic Review and Meta-Analysis by Tal Levit, Oluwatobi Olaiya, Declan C.T. Lavoie, Ronen Avram and Christopher J. Coroneos in Plastic Surgery

sj-tif-4-psg-10.1177_22925503251336253 - Supplemental material for The Use of Negative Pressure Wound Therapy for Breast Surgeries: A Systematic Review and Meta-Analysis Supplemental material, sj-tif-4-psg-10.1177_22925503251336253 for The Use of Negative Pressure Wound Therapy for Breast Surgeries: A Systematic Review and Meta-Analysis by Tal Levit, Oluwatobi Olaiya, Declan C.T. Lavoie, Ronen Avram and Christopher J. Coroneos in Plastic Surgery

sj-docx-5-psg-10.1177_22925503251336253 - Supplemental material for The Use of Negative Pressure Wound Therapy for Breast Surgeries: A Systematic Review and Meta-Analysis Supplemental material, sj-docx-5-psg-10.1177_22925503251336253 for The Use of Negative Pressure Wound Therapy for Breast Surgeries: A Systematic Review and Meta-Analysis by Tal Levit, Oluwatobi Olaiya, Declan C.T. Lavoie, Ronen Avram and Christopher J. Coroneos in Plastic Surgery
