# Supplementary material for: The Use of Negative Pressure Wound Therapy for Breast Surgeries: A Systematic Review and Meta-Analysis
Source: Plast Surg (Oakv). 2025 May 20;34(2):185–200. doi: 10.1177/22925503251336253 (PMC12116506; doi:10.1177/22925503251336253)
Supplement: sj-docx-1-psg-10.1177_22925503251336253 - Supplemental material for The Use of Negative Pressure Wound Therapy for Breast Surgeries: A Systematic Review and Meta-Analysis [file sj-docx-1-psg-10.1177_22925503251336253.docx]

**MEDLINE (July 10, 2023)**

Ovid MEDLINE(R) ALL <1946 to July 05, 2023>

1 exp Breast/ or exp Breast Implantation/ or exp Breast Implants/ or exp Breast Neoplasms/ or exp Breast Diseases/ or exp Mastectomy/ or exp Mammaplasty/ or exp Surgical Flaps/ or exp Tissue Expansion Devices/ 451959

2 (breast adj3 (surg* or microsurg* or recon* or augment* or reduc* or implant* or lift* or cancer* or transplant* or conserv*)).mp. [mp=title, book title, abstract, original title, name of substance word, subject heading word, floating sub-heading word, keyword heading word, organism supplementary concept word, protocol supplementary concept word, rare disease supplementary concept word, unique identifier, synonyms, population supplementary concept word, anatomy supplementary concept word] 383827

3 ((free or tissue or perfor* or surg*) adj3 (flap or transfer or graft)).mp. [mp=title, book title, abstract, original title, name of substance word, subject heading word, floating sub-heading word, keyword heading word, organism supplementary concept word, protocol supplementary concept word, rare disease supplementary concept word, unique identifier, synonyms, population supplementary concept word, anatomy supplementary concept word] 60598

4 (Mastect* or mammect* or mammapl* or mastopex* or lumpect*).mp. [mp=title, book title, abstract, original title, name of substance word, subject heading word, floating sub-heading word, keyword heading word, organism supplementary concept word, protocol supplementary concept word, rare disease supplementary concept word, unique identifier, synonyms, population supplementary concept word, anatomy supplementary concept word] 58504

5 1 or 2 or 3 or 4 607613

6 exp negative-pressure wound therapy/ 3956

7 (negative pressure adj3 (wound* or therap* or dress* or topic* or drain* or seal*)).mp. [mp=title, book title, abstract, original title, name of substance word, subject heading word, floating sub-heading word, keyword heading word, organism supplementary concept word, protocol supplementary concept word, rare disease supplementary concept word, unique identifier, synonyms, population supplementary concept word, anatomy supplementary concept word] 5948

8 (NPWT* or NPT* or ciNPT or PICO or VAC or PREVENA).mp. [mp=title, book title, abstract, original title, name of substance word, subject heading word, floating sub-heading word, keyword heading word, organism supplementary concept word, protocol supplementary concept word, rare disease supplementary concept word, unique identifier, synonyms, population supplementary concept word, anatomy supplementary concept word] 14223

9 (vacuum* adj3 (therap* or drain* or seal* or dress* or clos*)).mp. [mp=title, book title, abstract, original title, name of substance word, subject heading word, floating sub-heading word, keyword heading word, organism supplementary concept word, protocol supplementary concept word, rare disease supplementary concept word, unique identifier, synonyms, population supplementary concept word, anatomy supplementary concept word] 3926

10 6 or 7 or 8 or 9 19688

11 5 and 10 1201

**EMBASE (July 10, 2023)**

Embase <1974 to 2023 July 05>

1 exp breast/ or exp breast disease/ or exp breast surgery/ or exp breast reconstruction/ or exp breast prosthesis/ or breast tissue expander/ or exp surgical flaps/ 793665

2 (breast adj3 (surg* or microsurg* or recon* or augment* or reduc* or implant* or lift or cancer* or transplant* or conserv*)).mp. [mp=title, abstract, heading word, drug trade name, original title, device manufacturer, drug manufacturer, device trade name, keyword heading word, floating subheading word, candidate term word] 685807

3 ((free or tissue or perfor* or surg*) adj3 (flap or transfer or graft)).mp. [mp=title, abstract, heading word, drug trade name, original title, device manufacturer, drug manufacturer, device trade name, keyword heading word, floating subheading word, candidate term word] 90905

4 (Mastect* or mammect* or mammapl* or mastopex* or lumpect*).mp. [mp=title, abstract, heading word, drug trade name, original title, device manufacturer, drug manufacturer, device trade name, keyword heading word, floating subheading word, candidate term word] 83275

5 1 or 2 or 3 or 4 938367

6 exp vacuum assisted closure/ or vacuum assisted closure device/ 10518

7 (negative pressure adj3 (wound* or therap* or dress* or topic* or drain* or seal*)).mp. [mp=title, abstract, heading word, drug trade name, original title, device manufacturer, drug manufacturer, device trade name, keyword heading word, floating subheading word, candidate term word] 5939

8 (NPWT* or NPT* or ciNPT or PICO or VAC or PREVENA).mp. [mp=title, abstract, heading word, drug trade name, original title, device manufacturer, drug manufacturer, device trade name, keyword heading word, floating subheading word, candidate term word] 20475

9 (vacuum* adj3 (therap* or drain* or seal* or dress* or clos*)).mp. [mp=title, abstract, heading word, drug trade name, original title, device manufacturer, drug manufacturer, device trade name, keyword heading word, floating subheading word, candidate term word] 13005

10 6 or 7 or 8 or 9 30245

11 5 and 10 1922

**CINAHL (July 10, 2023)**

S1 (MH "Breast+") OR (MH "Breast Diseases+") OR (MH "Breast Reconstruction") OR (MH "Mastectomy+") OR (MH "Breast Implants") OR (MH "Tissue Expansion+") OR (MH "Surgical Flaps+") 112983

S2 (breast) N3 (surg* or microsurg* or recon* or augment* or reduc* or implant* or lift or cancer* or transplant* or conserv*) 101880

S3 ((free or tissue or perfor* or surg*) N3 (flap or transfer or graft)) 18640

S4 (Mastect* or mammect* or mammapl* or mastopex* or lumpect*) 11528

S5 S1 OR S2 OR S3 OR S4 148755

S6 (MH "Negative Pressure Wound Therapy") 3040

S7 (negative pressure) N3 (wound* or therap* or dress* or topic* or drain* or seal*) 3701

S8 (NPWT* or NPT* or ciNPT or PICO or VAC or PREVENA) 4309

S9 (vacuum*) N3 (therap* or drain* or seal* or dress* or clos*) 1061

S10 S6 OR S7 OR S8 OR S9 OR S10 7213

S11 S5 AND S11 410

**Web of Science (July 10, 2023)**

1: TS=("Breast" OR "Breast Disease*" OR "Breast Recon*" OR "Mastect*" OR "Breast Implant*" OR "Tissue Expan*" OR "Surg* Flap*") Results: 822363

2: TS=((breast) NEAR/3 (surg* OR microsurg* OR recon* OR augment* OR reduc* OR implant* OR lift OR cancer* OR transplant* OR conserv*)) Results: 633421

3: TS=((free OR tissue OR perfor* OR surg*) NEAR/3 (flap OR transfer OR graft)) Results: 129010

4: TS=(Mastect* OR mammect* OR mammapl* OR mastopex* OR lumpect*) Results: 37584

5: #4 OR #3 OR #2 OR #1 Results: 944935

6: TS=("Negative Pressure Wound Therapy" OR "Negative Pressure Therapy" OR "Vacuum Assisted Clos*") Results: 5680

7: TS=(negative pressure NEAR/3 (wound* OR therap* OR dress* OR topic* OR drain* OR seal*)) Results: 8515

8: TS=(NPWT* OR NPT* OR ciNPT OR PICO OR VAC OR PREVENA) Results: 26160

9: TS=((vacuum*) NEAR/3 (therap* OR drain* OR seal* OR dress* OR clos*)) Results: 7949

10: #9 OR #8 OR #7 Results: 38138

11: #10 AND #5 Results: 1342

**CENTRAL (July 10, 2023)**

#1 MeSH descriptor: [Breast] explode all trees 1441

#2 MeSH descriptor: [Breast Diseases] explode all trees 18360

#3 MeSH descriptor: [Mammaplasty] explode all trees 471

#4 MeSH descriptor: [Mastectomy] explode all trees 2451

#5 MeSH descriptor: [Breast Implants] explode all trees 85

#6 MeSH descriptor: [Tissue Expansion Devices] explode all trees 42

#7 MeSH descriptor: [Surgical Flaps] explode all trees 1600

#8 ((breast NEXT/3 (surg* OR microsurg* OR recon* OR augment* OR reduc* OR implant* OR lift OR cancer* OR transplant* OR conserv*))):ti,ab,kw 43733

#9 (((free OR tissue OR perfor* OR surg*) NEXT/3 (flap OR transfer OR graft))):ti,ab,kw 3413

#10 ((Mastect* OR mammect* OR mammapl* OR mastopex* OR lumpect*)):ti,ab,kw 6661

#11 #1 OR #2 OR #3 OR #4 OR #5 OR #6 OR #7 OR #8 OR #9 OR #10 51110

#12 MeSH descriptor: [Negative-Pressure Wound Therapy] explode all trees 347

#13 ((negative pressure NEXT/3 (wound* OR therap* OR dress* OR topic* OR drain* OR seal*))):ti,ab,kw 1156

#14 (NPWT* OR NPT* OR ciNPT OR PICO OR VAC OR PREVENA):ti,ab,kw 2183

#15 ((vacuum*) NEXT/3 (therap* OR drain* OR seal* OR dress* OR clos*)):ti,ab,kw 852

#16 #12 #13 OR #14 OR #15 2677

#17 #11 AND #16 147
