# Supplementary material for: The Use of Negative Pressure Wound Therapy for Breast Surgeries: A Systematic Review and Meta-Analysis
Source: Plast Surg (Oakv). 2025 May 20;34(2):185–200. doi: 10.1177/22925503251336253 (PMC12116506; doi:10.1177/22925503251336253)
Supplement: sj-docx-2-psg-10.1177_22925503251336253 - Supplemental material for The Use of Negative Pressure Wound Therapy for Breast Surgeries: A Systematic Review and Meta-Analysis [file sj-docx-2-psg-10.1177_22925503251336253.docx]

| Study ID | Procedure | Intervention | Site | Control |
| --- | --- | --- | --- | --- |
| Randomized Controlled Trials | | | | |
| Galiano  2018 | Bilateral reduction mammaplasty | PICO (-80mmHg)  removed POD7 | Breast | Sterile adhesive strips |
| Muller-Sloof  2018 | Breast reconstruction with DIEP or PAP flap | PREVENA (-125mmHg)  removed POD5-7 | Donor | Sterile adhesive strips |
| Tanaydin  2018 | Bilateral reduction mammaplasty | PICO (-80mmHg)  removed POD7 | Breast | Sterile adhesive strips |
| Fogacci  2020 | Quadrantectomy, mastectomy, or breast reconstruction | PICO  removed POD7 | Breast | Tissue-non-tissue plaster |
| Larsen  2020 | Mastectomy with sentinel lymph node biopsy or axillary clearance | PICO (-80mmHg)  removed POD7 | Breast | Surgical micropore paper tape |
| Timmermans  2021 | Bilateral gender-affirming mastectomy | PICO (-80mmHg)  removed POD7 | Breast | Sterile adhesive strips |
| Muller-Sloof  2022 | Breast reconstruction with DIEP flap | PREVENA (-125mmHg)  removed POD5-7 | Donor | Sterile adhesive strips |
| Pieszko  2023 | Mastectomy with immediate implant or expander-based breast reconstruction | Avelle (-80mmHg)  removed POD7 | Breast | Sterile adhesive tape |
| Observational Trials | | | | |
| Pellino  2014 | Variety of breast surgeries | PICO (-80mmHg)  removed POD7 | Breast | Absorbent dressings and gauze |
| Kim  2016 | Immediate expander-based breast reconstruction | (-125mmHg)  removed POD3 | Breast | Polyurethane foam dressing |
| Angspatt  2017 | Breast reconstruction with latissimus dorsi flap | RENASYS (-80 to -125 mmHg) removed POD3 | Donor | Standard dressing |
| Ferrando  2018 | Variety of breast surgeries | PREVENA (-125mmHg)  removed POD7 | Breast | Sterile adhesive strips |
| Gabriel  2018 | Expander-based breast reconstruction | PREVENA (-125mmHg)  removed POD7 | Breast | Sterile adhesive strips |
| Fang  2020 | Breast reconstruction with immediate bipedicle DIEP flap | PREVENA  removed POD6 | Donor | Silver hydrocolloid dressing |
| Irwin  2020 | Mastectomy with immediate implant-based breast reconstruction | PICO (-80mmHg) | Breast | Opsite dressing with absorbent pad |
| Kim  2020 | Mastectomy with direct-to-implant breast reconstruction | PICO (-80 mmHg)  removed POD7 | Breast | Standard dressing |
| Savage  2020 | Bilateral reduction mammaplasty | PREVENA (-125mmHg)  removed POD7 | Breast | Adhesive fabric dressing and gauze |
| Siegwart  2020 | Breast reconstruction with abdominal based free flaps | Self-made device  (-120mmHg) | Donor | Sterile adhesive strips |
| De Rooij  2021 | Mastectomy or modified radical mastectomy | Avelle (-80mmHg)  removed POD7 | Breast | Standard dressing |
| Doval  2021 | Breast reconstruction with DIEP flap | PREVENA (-125mmHg) | Donor | Standard dressing |
| Johnson III  2021 | Bilateral reduction mammaplasty | PREVENA (-125mmHg)  removed POD7 | Breast | Standard dressing |
| Wang  2021 | Mastectomy and breast reconstruction with DIEP or MS-TRAM flap | PREVENA (-125mmHg)  removed POD5-7 | Donor | Hydrocolloid dressing |
| El Hawa  2022 | Gender-affirming mastectomy with free nipple graft | PREVENA (-125mmHg)  removed POD7-10 | Breast | Bolster dressing |
| Kang  2022 | Unilateral breast reconstruction with DIEP flap | PICO (-80mmHg)  removed POD10 | Donor | Film dressing |
| Limpiado  2022 | Breast reconstruction with abdominal based flaps | PREVENA (-125mmHg)  removed up to POD10 | Donor | Standard dressing |
| Ryu  2022 | Mastectomy with immediate implant-based breast reconstruction | PICO (-80mmHg)  removed POD7 | Breast | Foam dressing |
| Siegwart  2022 | Breast reconstruction with TMG flap | RENASYS (-120mmHg)  removed POD5 | Donor | Sterile adhesive strips |
| Al-Ishaq  2023 | Variety of breast surgeries | PICO (-80mmHg)  removed POD7 | Breast | Sterile adhesive strips |
| Munro  2023 | Breast reconstruction with DIEP flap | PREVENA (-125mmHg)  removed POD7 | Donor | Standard dressing |
| Ockerman  2023 | Lumpectomy with bilateral mammaplasty or mastopexy | PREVENA (-125mmHg)  removed POD7-14 | Breast | Standard dressing |
| Wareham  2023 | Breast conservation surgery with immediate volume displacement or replacement | PREVENA (-125mmHg) | Breast | Sterile adhesive strips and skin glue |

Abbreviations:

Avelle: Avelle™, Convatec, UK

DIEP: Deep inferior epigastric artery perforator flap

MS-TRAM: Muscle-sparing transverse rectus abdominis myocutaneous flap

Opsite: Opsite, Smith & Nephew, UK

PAP: Profunda artery perforator flap

POD: Post-operative day

PICO: PICO™, Smith & Nephew, UK

PREVENA: 3M™ PREVENA™ Therapy, 3M, USA

RENASYS: RENASYS™, Smith & Nephew, UK

TMG: Transverse myocutaneous gracilis flap
