# Supplementary figures and images for: The Use of Negative Pressure Wound Therapy for Breast Surgeries: A Systematic Review and Meta-Analysis
Source: Plast Surg (Oakv). 2025 May 20;34(2):185–200. doi: 10.1177/22925503251336253 (PMC12116506; doi:10.1177/22925503251336253)

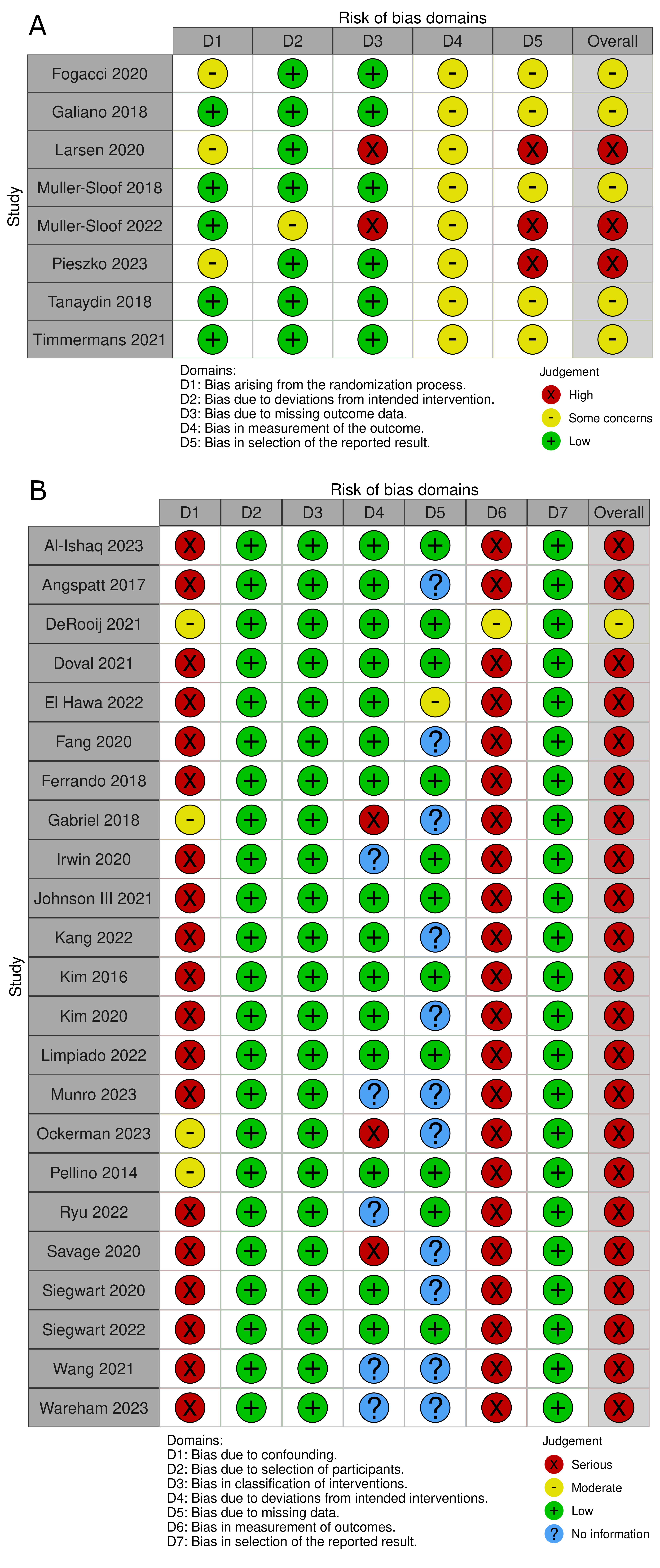

Supplement: sj-tif-3-psg-10.1177_22925503251336253 - Supplemental material for The Use of Negative Pressure Wound Therapy for Breast Surgeries: A Systematic Review and Meta-Analysis [file sj-tif-3-psg-10.1177_22925503251336253.tif]

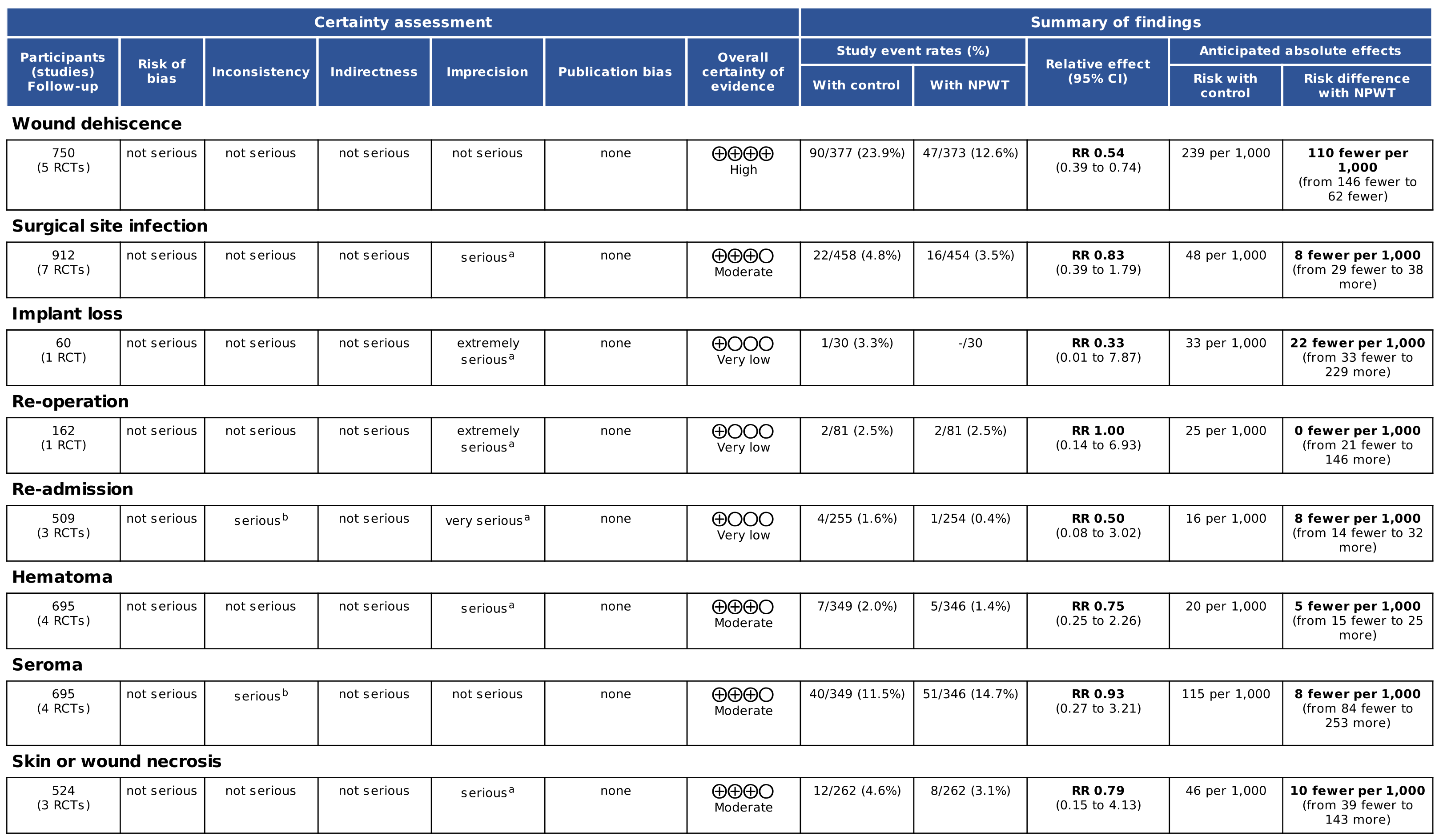

Supplement: sj-tif-4-psg-10.1177_22925503251336253 - Supplemental material for The Use of Negative Pressure Wound Therapy for Breast Surgeries: A Systematic Review and Meta-Analysis [file sj-tif-4-psg-10.1177_22925503251336253.tif]
